# Reproductive health problem among youths and associated factors in Dilla town, southern Ethiopia

**DOI:** 10.3389/frph.2025.1519024

**Published:** 2025-04-28

**Authors:** Seblewongel Gebretsadik Sertsewold, Getachew Assefa Zenebe, Amanuel Yosef Gebrekidan

**Affiliations:** ^1^ Department of Public Health, Asirat Woldeyes Health Science Campus, Debrebirhan University, Debrebirhan, Ethiopia; ^2^ School of Public Health, College of Health Sciences and Medicine, Dilla University, Dilla, Ethiopia; ^3^ School of Public Health, College of Health Sciences and Medicine, Wolaita Sodo University, Wolaita, Ethiopia

**Keywords:** reproductive health, southern Ethiopia, problem, youth, factors

## Abstract

**Introduction:**

Adolescents are defined by the World Health Organization as people aged 10–19, youth 15–24, and young people 10–24. Youth are a demographic that experiences life-threatening physical, emotional, mental, and social changes. Most young individuals are therefore more vulnerable to reproductive health problems than adults for a number of reasons.

**Methods:**

A community-based cross-sectional study was done in Dilla town between January and March 2024 with 536 youths. A survey of houses with young people was followed by the use of a systematic sampling technique. Data was gathered using pretested and structured questionnaires. Coded and entered into EPI INFO version 7 after being verified for completeness, it was then exported to SPSS version 22 for analysis. Both multivariate and bivariate logistic regression were performed. Variables with a *p*-value <0.2 in bi-variable logistic regression were moved to multivariable logistic regression, and variables with a *p*-value of <0.05 in multivariable logistic regression were considered statistically significant.

**Result:**

A total of 493 youths participated in this study, with a response rate of 92%. Among them, 271 (55%) of them were under the age group of 21–25 years old, 225 (45.6%) were females, and 214 (43.4%) were protestants. In addition, 287 (58.2%) said that they are ashamed to discuss sexual issues, and a few respondents, 158 (32%), had multiple sexual partners. Most of the respondents (92.1%, 87.4%, 100%, and 72.6%) reported that they had no history of khat chewing, cigarette smoking, shisha smoking, or drinking alcohol, respectively. The overall youth reproductive health problem in Dilla town is 66.1% (95% CI: 62.5–70.4). Being in the age category 21–25 (AOR: 8.52; 95% CI: 4.61–15.76), employed (AOR: 2.12; 95% CI: 1.09–4.13), ashamed to discuss sexual issues (AOR: 6.66; 95% CI: 3.79–11.71), having multiple sexual partners (AOR: 2.01; 95% CI: 1.08–3.73), not using condoms (AOR: 4.05; 95% CI: 2.31–7.11), health facility inconvenience (AOR: 3.2; 95% CI: 1.85–5.55), and drinking alcohol (AOR: 2.28; 95% CI: 1.29–3.99) were found to be significantly associated with youth reproductive health problems.

**Conclusion and recommendation:**

Healthcare facilities should provide comprehensive, age-appropriate, and accurate sexual education as part of specialized reproductive health services for youth that prioritize privacy and nonjudgmental treatment. In addition, screening and early detection of RH problems and engaging in community outreach programs are some of the recommended activities.

## Introduction

The World Health Organization defines youth as people between the ages of 15 and 24 who exhibit substantial physiological, psychological, and social changes that put their lives at risk. According to estimates, youth between the ages of 15 and 24 make up 17.0% of the world's population, 20.0% of Sub-Saharan Africa, and 17.9% of Ethiopia's population (1, 2).

Around the world, young people are dealing with a variety of sexual and reproductive health issues, including unintended pregnancy, unsafe abortion, sexually transmitted infections, and human immunodeficiency virus. Young people do not frequently require specialized medical care despite being misunderstood as being in good health. Globally, there are 1.8 billion young people; the majority live in low- and middle-income nations. Young people in low and middle-income nations, numbering 1.3 million, are reported to die every year from preventable causes despite being the healthiest segment of the population (2, 3).

Young people did not have access to proper reproductive health information and services, and they underutilized these services when they were provided. The challenges linked with youth reproductive health have become more serious and complex as a result of this predicament (4). Despite the comprehensive knowledge of HIV and other Reproductive Health (RH) problems increasing around the world, many young people do not have the information or means to protect themselves from these problems. The lack of information about risky sexual behaviors significantly impacts young people, making them more vulnerable to the challenges of early marriage, unwanted pregnancy, HIV, STIs, and other reproductive health problems (5–7).

The majority of young people begin sexual activity throughout their adolescence with several partners before their eighteenth birthday. It is estimated that 30–35 million abortions are performed globally each year, with about half of them being illegal. Teenagers account for a significant part of all abortions. The prevalence of STIs is higher among young people aged 15–24 in Sub-Saharan Africa than in any other age group (8–10).

Nearly 6,000 young individuals between the ages of 15 and 24 get infected with HIV every day around the world. However, only a small percentage of individuals are aware that they are sick. There is a solution to stop HIV/AIDS from spreading, focusing on young people. Today, more than half of those newly infected with HIV are between the ages of 15 and 24 (11, 12).

Early marriage is frequent in many parts of the world, but it is most prevalent in Africa and South Asia. In Nigeria, 76% of females get married by the age of 18, compared to 50% in India. In Nepal, 19% of girls are married before they are 15, and 60% are married by the age of 18 (13).

Early marriage, which roughly corresponds to menarche, and early frequent childbearing with a subsequent abortion, which is often dangerous, or carrying the fetus to full term, which limits their possibilities for socioeconomic growth, are common in Ethiopia. According to findings, children born to teenage mothers have a significantly higher risk of dying early. Unwanted pregnancy among teens was reported to be 30.1% in Gondar, Ethiopia. Unwanted pregnancy among teens was reported to be 30.1% in Gondar, Ethiopia (14–16).

Young people in Ethiopia face complex and interconnected reproductive health issues, which are worsened by societal, economic, environmental, and cultural behaviors. The nature of the issues necessitates a multi-sectorial and integrated approach to teenage reproductive health. Apart from reproductive health, Ethiopian teenagers face a variety of issues, including poverty and traditional and cultural beliefs. Their material, social, and reproductive needs have not received the attention they deserve as a group (6, 17).

People commonly motivated their sexual interactions with youths by offers of monetary gain, gifts, career positions, or promises to send money abroad. This is most common among considerably elderly males, and there are no cultural consequences for it. According to their description, these males simultaneously satisfy the girl's economic demand while also gaining the advantage of being young and seemingly disease-free sex clients (18).

Several factors can affect the health of the adolescent population, including age, cultural views on early marriage, cultural restrictions on contraception use, a lack of understanding about the risks of unprotected sexual activities, and insufficient life skills needed to practice safer sexual behaviors. In many low- and middle-income countries, youth reproductive health problems are largely confounded by limited access to reproductive health treatments, inadequate service provision, and a lack of awareness among unmarried young people (19).

The majority of emerging countries began with national youth strategies. Preventive programs and strategic plans for young generations' sexual and reproductive health problems or issues are few and far between in most developing countries, but national youth policies, programs, and strategies that specifically address and meet young people's sexual and reproductive health needs are common (9).

To address youth reproductive health issues, the Ethiopian Ministry of Health has devised adolescent and youth health strategies. Despite efforts made to increase access to adolescent reproductive health care, utilization remains low, and the youth population is disproportionately affected by reproductive health issues. Researchers conducted various studies on the use of youth reproductive health services and associated factors in Ethiopia. However, there is a paucity of data on youth reproductive health issues, service preferences, and other related aspects. In this context, a study was conducted to assess reproductive health problems, service preferences, and related factors among teenagers in Dilla town, southern Ethiopia.

## Methods and materials

### Study design and setting

**Figure 1:**
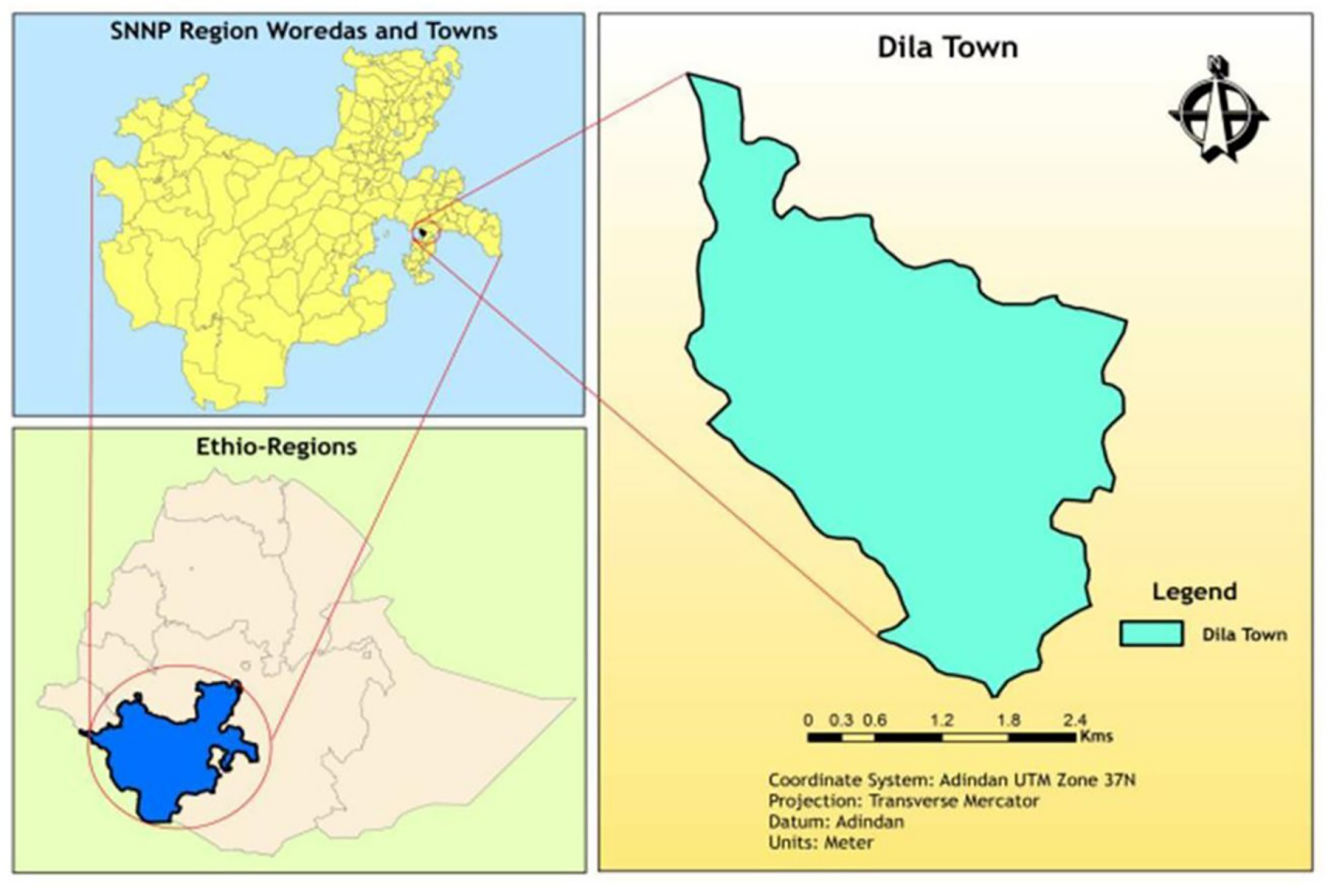
Map of Dilla town, Ethiopia (20).

A community-based cross-sectional study was conducted from January to March 2024 in Dilla town, which is located in southern Ethiopia at a distance of 359 km from the capital city of Addis Ababa at an altitude of 1,500–3,000 m above sea level. Its climatic condition is “Woynadega”. Of Dilla Town's approximately 94,148 residents, 34,452 are thought to be young people between the ages of 15 and 24. There are nine kebeles and three sub-cities in the town. Dilla has 1 referral hospital, 2 health centers, 15 private clinics, 18 drugstores, and 4 pharmacies (Figure 1).

### Source and study population

The source population consisted of all youths living in Dilla town, while the study population consisted of youths from selected kebeles during the data collection period. Youths who were unable to communicate at the time of the study were excluded.

### Sample size determination and sampling procedure

The sample size was determined using a single population proportion formula with the following assumption: a significance level of 95%, a 4% margin of error, and a magnitude of youth reproductive health problems of 28.3% from the study in Lay Gayint district, Amhara region (20), and a 10% non-response rate.

$$n\;=\;(Z\;\alpha /2{)}^{2}\times \;p\;(q)/d^{2}$$

where:

*n* = required sample size, Z *α*/2 = confidence level (1.96)
p = population proportion (0.283), q = 1-p (0.717)
**d** =margin of error (0.04)
*n* = (1.96)^2^ × (0.283) × (0.717) (0.04)^2^
*n* = 3.842 × 0.203/0.0016
*n* = 0.779/0.0016
*n* = 488 + 10% non-response rate
*n* = 536

Thus, according to the above assumptions and the given formula, the total calculated sample size was 536. Using the lottery method, one sub-city and 4 kebeles were selected from Dilla town. The survey was done to identify households having youths in each selected kebele. The calculated sample size was proportionally allocated to the selected kebele, and then the required samples were taken by using a systematic random sampling technique.

### Variables and measurements

Youth reproductive health problem was the dependent variable. A participant is considered to have a reproductive health problem if he/she experiences at least one of the major reproductive health problems (unplanned pregnancy, abortion, early sexual initiation, and symptoms of sexually transmitted infections). The independent variables were sex, age, marital status, religion, educational status, occupational status, family condition, parent's job status, family monthly income, pocket money, being ashamed to discuss sexual issues, having multiple sexual partners, using condoms during sexual intercourse, peer influence, convenience of health facilities, utilization of youth reproductive health services, premarital sexual intercourse, contraceptive use, and substance use-related variables (khat chewing, cigarette smoking, shisha smoking, and drinking alcohol) (20–22).

Substance use was measured by asking whether youths were taking any of the three commonly used psychoactive substances: alcohol, cigarettes, and khat in the past 30 days (23).

Early sexual initiation was considered if the participants experienced sexual initiation before 18 years of age (20).

### Data collection instruments and quality control

The interviewer-administered questionnaire was developed from previous literature and was prepared in English, then translated to Amharic, and finally back to English to maintain its consistency. The questionnaire was composed of a consent form and questions in three major categories.

Before conducting the actual study, the questionnaire was pretested on 5% (27) of the sample size among youths in Wonago town. After taking necessary corrections and amendments, training was given for six data collectors with a diploma background in health science for 2 days. During the data collection, regular monitoring and supervision were done to verify the consistency, quality, clarity, and completeness of the data. Appropriate coding and data cleaning were carried out to ensure data quality.

### Data management and analysis

Data was checked for completeness, coded, and entered into EPI INFO version 7, and then exported to SPSS version 22 for analysis. Descriptive statistics were done, and the results were presented with tables based on the nature of the variables.

Bivariable and multivariable logistic regression was done. The candidate variables with a *p*-value <0.2 in bivariable logistic regression were moved to multivariable logistic regression to identify their significance with dependent variables, and those variables that affected youth reproductive health were identified by considering a *p*-value <0.05 with a 95% CI. The Hosmer-Lemeshow goodness-of-fit test was done to assess the adequacy of the logistic regression model, which indicates that the model provides a good fit to the data, as a *p*-value >0.05 suggests no significant difference between observed and predicted values.

## Results

### Socio-demographic characteristics of the participants

A total of 493 youths participated in this study, with a response rate of 92%. Among them, 271 (55%) were under the age group of 21–25 years old, 225 (45.6%) were females, and 214 (43.4%) were protestants. Almost all 484 (98.2%) of the participants started school, and 243 (49.3%) had college and above educational status. Just 40 (8.1%) of the participants had a job, and 432 (87.6%) of the participants were single. For everyday expenses, 323 participants, or 65.5%, received pocket money, and 422 participants, or 85.6%, derived their income from their parents. 326 (66.1%) of the participant's family live together, and 224 (45.4%) of the participant's family both work outside the home (Table 1).

**Table 1:** Socio-demographic characteristics of youths in the study of reproductive health problem among youths and associated factors in Dilla town, southern Ethiopia.

Variables	Category	Frequency	Percent (%)
Age	15–20	222	45
	21–25	271	55
Sex	Male	268	54.4
	Female	225	45.6
Religion	Orthodox	206	41.8
	Catholic	7	1.4
	Protestant	214	43.4
	Muslim	66	13.4
Education status	Only read and write	7	1.4
	1–8	69	14
	9–12	174	35.3
	College and above	243	49.3
Marital status	Unmarried	432	87.6
	Married	61	12.4
Family condition	Live together	326	66.1
	Separated	167	33.9
Occupation	Student	370	75.1
	Employed	123	24.9
Parents job status	Both works outside	224	45.4
	Only my father work outside	229	46.5
	Only my mother work outside	40	8.1
Get pocket money	Yes	323	65.5
	No	170	34.5
Source of pocket money	Relative	15	3.0
	Spouse	16	3.2
	Parents	422	85.6
	Skip	40	8.1

### Reproductive health-related characteristics of respondents

Among participants, 287 (58.2%) said that they were ashamed to discuss sexual issues, and a few respondents, 158 (32%), had multiple sexual partners. More than half of respondents, 308 (62.5%), didn't use condoms during their recent sexual intercourse, and 213 (43.2%) of respondents reported that there was peer influence on sexually related issues. Nearly half of respondents (217, 44%) reported that health facilities were convenient for youths. Only a few youths, 43 (8.7%), use youth reproductive health services; 41 (8.3%) had premarital sexual intercourse; and 120 (24.3%) use modern contraceptive methods (Table 2).

**Table 2:** Reproductive health characteristics of youths in the study of reproductive health problem among youths and associated factors in Dilla town, southern Ethiopia.

Variables	Category	Frequency	Percent (%)
Ashamed to discuss about sexual issues	Yes	287	58.2
	No	206	41.8
Multiple sexual partner	Yes	158	32
	No	335	68
Condom use	Yes	219	44.4
	No	274	55.6
Peer influence on sexual issues	Yes	213	43.2
	No	280	56.8
Convenience of health facility	Yes	217	44
	No	276	56
Use youth reproductive health service	Yes	43	8.7
	No	450	91.3
Premarital sexual intercourse	Yes	41	8.3
	No	452	91.7
Use of modern contraceptives	Yes	120	24.3
	No	373	75.7

### Substance use-related characteristics of respondents

Most of the respondents (92.1%, 87.4%, 100%, and 72.6%) reported that they had no history of chewing khat, smoking cigarettes, smoking shisha, or drinking alcohol, respectively (Table 3).

**Table 3:** Substance use related characteristics of youths in the study of reproductive health problem among youths and associated factors in Dilla town, southern Ethiopia.

Variables	Category	Frequency	Percent (%)
Khat chewing	Yes	39	7.9
	No	454	92.1
Smoking cigarette	Yes	62	12.6
	No	431	87.4
Alcohol drinking	Yes	275	55.8
	No	218	44.2

### Preference for youth's reproductive health services

According to a survey on youth preference for reproductive health care, 238 (48.3%) of respondents said they would rather see a private health provider, and 443 (89.9%) said they did not use the current health facilities for their reproductive health requirements. More than half of the respondents (296, or 60%) wanted the service to take place during regular working hours at the health facility, 262 (53.1%) wanted a discounted rate, 394 (79.9%) wanted the service provider to be young and of the same sex, and 274 (55.6%) wanted the service to be in the town center.

According to the assessment, 374 (75.9%), 337 (68.4%), 331 (67.1%), 491 (99.6%), and 475 (96.3%) of the total respondents said they would prefer to participate in youth health information centers, receive sexual education, receive family planning services, receive partner relationship counseling, and receive information and education on HIV/AIDS and STDs, respectively (Table 4).

**Table 4:** Preference of youth's reproductive health service in the study of reproductive health problem among youths and associated factors in Dilla town, southern Ethiopia.

Variable	Category	Frequency	Percent (%)
Utilize existing health institution	Yes	50	10.1
	No	443	89.9
Prefer to be served	Government health institution	195	39.6
	Private health institution	238	48.3
	FGAE clinic	26	5.3
	School clinic	34	6.9
Convenient time to use service	In usual working hour	296	60
	In hours when other users are not found	197	40
Preference in service fee	At usual rate	33	6.7
	With discount	262	53.1
	Free of charge	198	40.2
Preference of service provider	Young provider of the same sex	394	79.9
	Young provider of any sex	30	6.1
	Adult provider of the same sex	28	5.7
	Any provider	41	8.3
Preference of location	Anywhere out of resident area	219	44.4
	In the center of the town	274	55.6
Preference to have sex education	Yes	374	75.9
	No	66	13.4
	I don't know	53	10.8
Preference to have family planning service	Yes	337	68.4
	No	101	20.5
	I don't know	55	11.2
Preference to have partner relation guidance	Yes	331	67.1
	No	94	19.1
	I don't know	68	13.8
Preference to have an information and education on STD and HIV/AIDS	Yes	491	99.6
	I don't know	2	0.4
Preference to have youth health information center	Yes	475	96.3
	No	6	1.2
	I don't know	12	2.4

### Youth reproductive health problem

In Dilla town, the prevalence of youth reproductive health problems is 66.1% (95% CI: 62.5–70.4). In terms of beginning sexual activity, 275 people (55.7%) did so during the data collection period, and 198 people (40.2%) did so prior to turning 18 years old. Of the respondents, only 140 (28.4%) experienced an unintended pregnancy, and 49 (9.9%) had an abortion. STI symptoms were present in nearly half of the 200 respondents (40.6%) (Table 5).

**Table 5:** Youth reproductive health problems in Dilla town, southern Ethiopia.

Variables	Category	Frequency	Percent (%)
Initiation of sexual intercourse	Above 18 years old	295	59.8
	Less than 18years old	198	40.2
Abortion	Yes	49	9.9
	No	444	90.1
Unplanned pregnancy	Yes	140	28.4
	No	353	71.6
STD symptom	Yes	200	40.6
	No	293	59.4
Youth reproductive health problem	Yes	326	66.1
	No	167	33.9

### Factors related to youth reproductive health problems

In a bivariable analysis, the following factors were associated with youth reproductive health problems at a *P*-value <0.2: age, family status, occupation, multiple sexual partners, condom use, health facility convenience, being ashamed to discuss sexual issues, alcohol drinking, and the use of contraceptives.

Multivariable analysis revealed that youth reproductive health problems were significantly associated with age, occupation, condom use, having multiple sexual partners, being ashamed to discuss sexual issues, alcohol drinking, and health facility convenience (*P*-value <0.05). Model fitness was checked by the Hosmer and Lemeshow goodness of fit test, which was a *p*-value of 0.18, indicating that the model provides a good fit to the dataset.

Youths aged 21–25 years had 8.52 (AOR: 8.52; 95% CI: 4.61–15.76) times higher chances of experiencing problems related to reproductive health than youths aged 15–20 years. Additionally, youths who were employed were 2.12 (AOR: 2.12; 95% CI: 1.09–4.13) times more likely to have reproductive health problems compared to students.

Moreover, youths who were ashamed to discuss sexual issues were 6.66 (AOR: 6.66; 95% CI: 3.79–11.71) times more likely to have reproductive health problems compared to their counterparts.

Youths who had multiple sexual partners were 2.01 (AOR: 2.01; 95% CI: 1.08–3.73) times more likely to have reproductive health-related problems. Besides, the odds of having reproductive health problems among youths who didn't use condoms during sexual intercourse were 4.05 (AOR: 4.05; 95% CI: 2.31–7.11) times more likely to have reproductive health problems compared to those who were not using them.

Youths who believed health facilities were inconvenient were 3.2 (AOR: 3.2; 95% CI: 1.85–5.55) times more likely to have reproductive health problems compared to their counterparts. On the other hand, youths who drink alcohol were 2.28 (AOR: 2.28; 95% CI: 1.29–3.99) times more likely to have reproductive health problems compared to youths who don't drink alcohol (Table 6).

**Table 6:** Factors affecting youth reproductive health problems in Dilla town, southern Ethiopia.

Variable	Category	Youth reproductive health problem		COR	AOR	*P*-value
		Yes	No			
Age	15–20	90	132	1	1	
	21–25	236	35	9.8 (6.34–15.43)	8.52 (4.61–15.76)	0.00*
Occupation	Student	234	136	1	1	
	Employed	92	31	1.73 (1.09–2.73)	2.12 (1.09–4.13)	0.02*
Family condition	Live together	196	130	1	1	
	Separated	130	37	2.33 (1.52–3.57)	1.23 (0.67–2.23)	0.5
Ashamed to discuss	Yes	242	45	7.81 (5.12–11.92)	6.66 (3.79–11.71)	0.00*
	No	84	122	1	1	
Multiple sexual partner	Yes	119	39	1.89 (1.24–2.88)	2.01 (1.08–3.73)	0.03*
	No	207	128	1	1	
Condom use	Yes	96	123	1	1	
	No	230	44	6.69 (4.41–10.18)	4.05 (2.31–7.11)	0.00*
Convenience of health facility	Yes	104	113	1	1	0.00*
	No	222	54	4.47 (2.99–6.66)	3.2 (1.85–5.55)	0.00*
Contraceptive use	Yes	68	52	1	1	
	No	258	115	1.72 (1.12–2.62)	1.42 (0.73–2.73)	0.3
Alcohol drinking	Yes	225	50	5.21 (3.47–7.82)	2.28 (1.29–3.99)	0.00*
	No	101	117	1	1	

*Significant at *P*-value <0.05.

## Discussion

According to this study, 66.1% of young people in Dilla town had reproductive health problems. Factors that were found to be significantly associated with youth reproductive health problems included age, occupation, being ashamed to discuss sexual issues, having multiple sexual partners, condom use, and health facility convenience.

Based on this study, youth reproductive health problems are higher compared to the study done on female high school students in a Lay Gayint district (28.3%) (20). This discrepancy might be due to male youths included in this study, who are greater in number compared to females. Even though reproductive health issues often focus on females, males are also vulnerable to higher-risk sexual behaviors. Some STIs are asymptomatic in males and easily overlooked, which leads to long-term complications and an increased transmission rate.

This study found that 40.6% of participants experienced STI symptoms, 28.4% experienced an unintended pregnancy, and 9.9% experienced an abortion. These rates are greater than those found in a study conducted among students at Ambo University in Central Ethiopia (22.8%, 5%, and 22.5%, respectively) (24). The disparity may result from variations in the study populations. Other study participants, besides students, could not have had access to health education, which caused them to make irresponsible sexual decisions and raised their risk of reproductive sexual problems.

This study found that youth reproductive health service use was only 8.7%, which is low compared to the studies done in Bahir Dar city (32%) (1), Dejen district (45%) (25), and Lay Gayint district (35.6%) (20). The possible explanation for the discrepancy might be due to the difference in economic and educational status among the study participants. Youth from low-income families and lower levels of education face barriers to accessing reproductive healthcare services and are less informed about sexual and reproductive health. Health service utilization plays a great role in improving and maintaining reproductive health. Regular access to and use of reproductive health services can prevent, diagnose, and treat various reproductive health issues.

Youths in the age group of 21–25 years old were 8.52 (AOR: 8.52; 95% CI: 4.61–15.76) times more likely to develop reproductive health problems. And this finding is in line with the study in the Lay Gayint district (20). Since this age group is a period of independence, less parental supervision and guidance may lead youths to be more vulnerable to reproductive health issues and risky sexual behaviors. Peer pressure and a desire to explore their sexuality may exacerbate this risk-taking.

Based on the findings, youths who are employed were 2.12 (AOR: 2.12; 95% CI: 1.09–4.13) times more likely to have reproductive health problems than students. This might be due to employed youths having an income, which may lead to lifestyle changes, including increased alcohol consumption, smoking, or unprotected sexual activity, which contribute to reproductive health issues.

Youths who were ashamed to discuss sexual issues were 6.66 (AOR: 6.66; 95% CI: 3.79–11.71) times more likely to have reproductive health problems than their counterparts, which is in line with the study in Lay Gayint (20). Youths who are not encouraged to talk about sexual concerns may not know enough about safe sexual practices and may be afraid to seek therapy, which could make their problems worse.

Youths who had multiple sexual partners were 2.01 (AOR: 2.01; 95% CI: 1.08–3.73) times more likely to have reproductive health problems than those who didn't have multiple sexual partners. This finding is in line with the study done in Lay Gayint (20). Having multiple sexual partners increases the rate of STI transmission and exposure to other reproductive health problems.

The odds of having reproductive health problems were 4.05 (AOR: 4.05; 95% CI: 2.31–7.11) times higher for youth who did not use condoms during sexual activity. Condoms are highly effective in reducing the transmission of different sexually transmitted diseases and unwanted pregnancies. An urgent report from the WHO regional office for Europe reveals that condom use among youths has declined significantly since 2014. This is putting young people at risk of various reproductive health problems (26).

Youths who perceived that health facilities were not convenient were 3.2 (AOR: 3.2; 95% CI: 1.85–5.55) more likely to have reproductive health problems than those who thought health facilities were convenient, which is in line with the study in Lay Gayint (20). If a health facility is difficult for people to get to and they are not interested in the services it offers, they might not want to visit.

Youths who drink alcohol were 2.28 (AOR: 2.28; 95% CI: 1.29–3.99) times more likely to have reproductive health problems compared to youths who don't drink alcohol. Alcohol consumption increases engagement in risky sexual behaviors, which has an impact on their reproductive health.

### Limitation of the study

The chicken-egg paradox is typical in cross-sectional studies, so it would be better if a qualitative study from the perspectives of other stakeholders was included. Since the study's findings are based on young people's self-reported data, social desirability bias may have an impact. Furthermore, this study is restricted to the study region and cannot be extrapolated to the entire southern country.

## Conclusion and recommendation

This study revealed that the magnitude of youth reproductive health problems was 66.1%, and age, occupation, shame of discussing sexual issues, multiple sexual partners, condom use, and health facility convenience were found to be significantly associated with youth reproductive health problems.

Government, national, and local health departments should develop policies and implement programs focused on youth reproductive health.

Healthcare facilities should provide comprehensive, age-appropriate, and accurate sexual education as part of specialized reproductive health services for youth that prioritize privacy and nonjudgmental treatment. In addition, screening and early detection of RH problems and engaging in community outreach programs are some of the recommended activities.

Educational institutions have to play a role in promoting healthy behavior by teaching students about responsible decision-making, promoting safe sexual practice, delaying sexual activity, and avoiding risky behaviors that lead to reproductive health problems. Additionally, support students who are at risk of early pregnancy, abuse, or other challenges.

By helping young people at home where social and cultural standards are upheld while fostering health, parents and community leaders can influence views in the community and play a significant part in breaking down cultural barriers. Programs for peer education, advocacy, and resources about youth reproductive health must be offered by NGOs and neighborhood organizations.

Researchers and academics should contribute to understanding youth reproductive health issues through studies and evaluations. Collaborative efforts among these stakeholders are essential to effectively address the reproductive health needs of youths. Further investigation with a large representative sample by incorporating other stakeholders is recommended.

## Data Availability

The raw data supporting the conclusions of this article will be made available by the authors, without undue reservation.
